# A case of mesothelial/monocytic incidental cardiac excrescence and literature review

**DOI:** 10.1186/1746-1596-5-40

**Published:** 2010-06-21

**Authors:** Zhong-Liang Hu, Hui Lü, Hong-Ling Yin, Ji-Fang Wen, Ou Jin

**Affiliations:** 1 Department of Pathology, Xiangya Medical School, Central South University, Changsha, Hunan Province, China; 2 Electron Microscope Lab, Xiangya Medical School, Central South University, Changsha, Hunan Province, China

## Abstract

Mesothelial/monocytic incidental cardiac excrescence (MICE) is a rare entity which is an amalgam of mesothelial cells, histiocytes, and fibrin, often found occasionally during cardiac valve replacement. We report a case in a 25-year-old Chinese female with serous mitral stenosis and patent foramen ovale. Routine and immunohistochemical stains and ultrastructure examination revealed the vegetation was predominantly composed of histocytes with scattered mesothelial cells. In fact nodular histiocytic/mesothelial hyperplasia (NHMH) is a similar lesion to MICE. MICE and NHMH could be unified, and NHMH may be a better choice.

## Background

Mesothelial/monocytic incidental cardiac excrescence (MICE) is a rare entity composed of a haphazard mixture of histiocytes, mesothelial cells, fibrin, adipocytes, and inflammatory cells without a vascular network or supporting stroma. Up to now its etiology remains unclear. There were two hypotheses that were proposed to explain how the mesothelial cells ''gain'' access into the intravascular compartment. However, neither the 'reactive'' theory nor "artifact" theory could fully elucidate the MICE reported. MICE may be just a reactive lesion which results from imflammation, mechanic irritation, or tumor. In fact nodular histiocytic/mesothelial hyperplasia (NHMH) is a similar entity to MICE. We propose to unify MICE and NHMH, and NHMH may be a better choice. Regardless of how MICE is formed, there is of clinical significance because it may be mistaken for malignancies.

## Case Presentation

A 25-year-old Chinese female coughed and had a fever 4 years ago. Since then she gradually felt chest distress, shortness of breath, nausea and weakness. In June 2006, she saw a doctor in Xiangya hospital out-patient clinic with pale complexion. Serious mitral stenosis with thickened leafs was found on a transthoracic echocardiogram accompanied by the dilation of left and right atrium and slightly dilation of right ventricle. She denied history of ischemic heart disease or prior cardiac catheterization. On physical examination, rough systolic ejection murmur and short diastolic rumble were heard at her apex area of heart. She was given an admission diagnosis of rheumatic heart disease. Following carefully examination, two mm split was observed in foramen ovale with left to right shunt. At surgery, "vegetation" was found free floating within the pericardial sac. However, the vegetation was invisible in the echocardiographic examination. Both the mitral valve and vegetation were carefully resected and sent for histological examination. She is alive and well 3 years after mitral valve replacement and foramen ovale closed.

**Figure 1:**
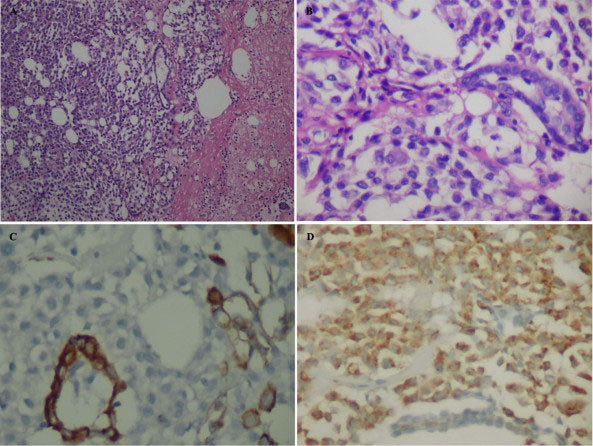
**Soft tissue vegetation in pericardial sac.** A, MICE is a mixture of histiocytes, mesothelial cells, fibrin, adipocytes, and inflammatory cells without a vascular network (Hematoxylin and eosin stain, × 100). B, Mesothelial gland-like structure are admixed with numerous histiocytes (Hematoxylin and eosin stain, ×200). C, The mesothelial cells are strongly immunoreactive for AE1/AE3 while the histiocytes are negative (DAB stain, ×400). D, The histiocytes are diffusely immunoreactive for CD68 while the mesothelial cells are negative (DAB stain, ×200).

**Figure 2:**
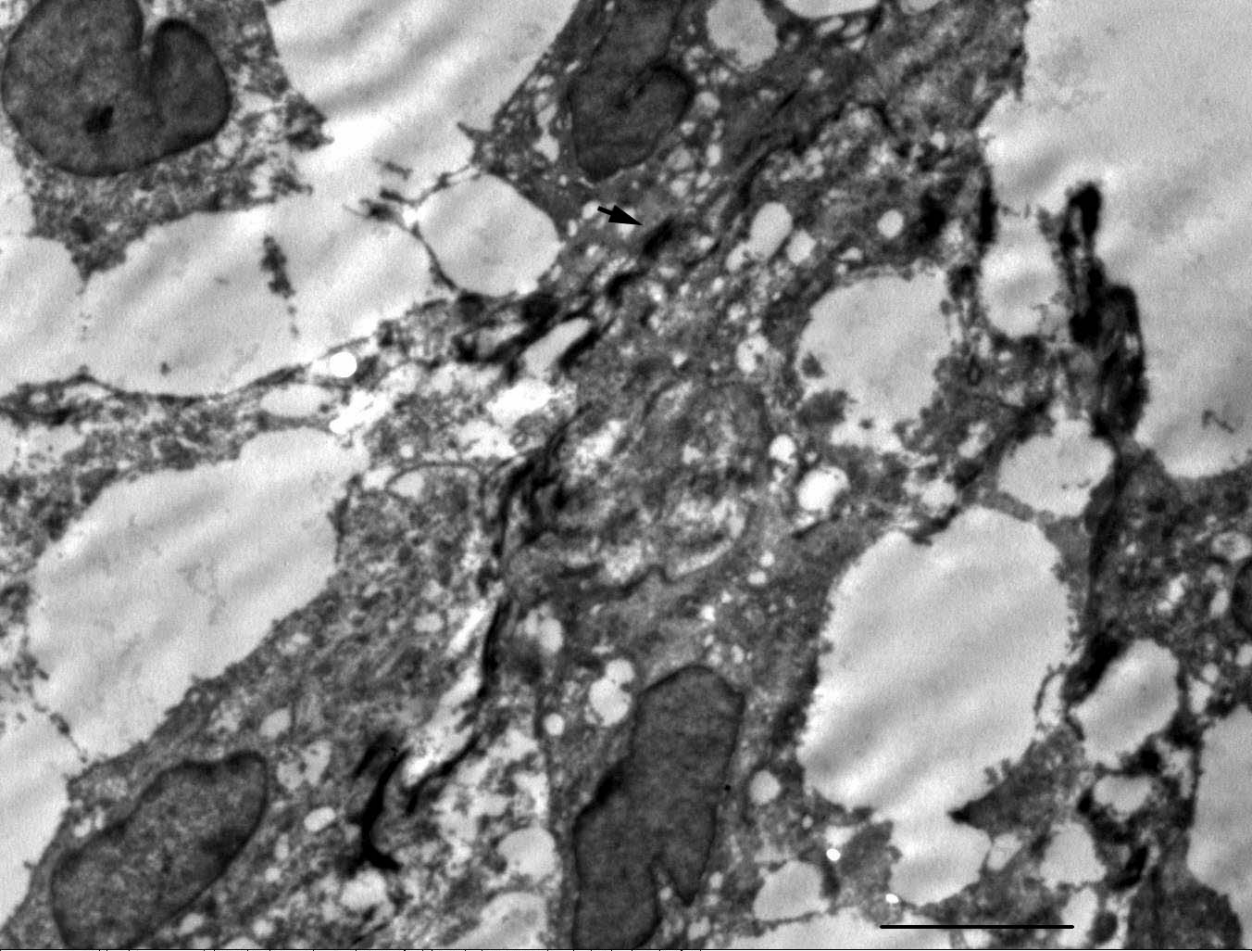
**Electron micrograph of MICE**. The mesothelial cells showed numerous desmosome-like structure as indicated by arrow. Bar: 5 μm.

Gross examination showed two recognizable slightly thickened valve leaflets, and an erythematous soft tissue fragment measuring 1.2 × 1 × 0.3 cm. Microscopically the soft tissue fragment consisted of epithelial strips arranged in glandlike structures admixed with numerous histiocytes, delicate fibrin strands, empty vacuoles suggestive of lipid droplets or adipocytes, with few neutrophils infiltration (Fig. 1A and 1B). Occasionally, multinucleated cells could be observed. But mitosis was not observed in either cell population. Supporting stroma, including blood vessels, was not observed. The epithelial strips composed of cuboidal to low columnar cells, showing strong membranous immunostaining for cytokeratin AE1/AE3 (Fig. 1C) and cytokeratin 5/6, strong nuclear and cytoplasmic immunostaining for calretinin, and nuclear immunostaining for WT-1, supporting its mesothelial origin. The histiocytic component showed intense cytoplasmic immunostaining for CD68 (Fig. 1D) and lysozyme. Neither of cell components was stained with antibodies against S-100, synaptophysin, chromogranin, and CD34. The epithelial cells contained desomosome-like structures and microvilli in ultrastructure (Fig. 2), while rich histiocytoid cells showed renal-shape nuclei and a few cytoplasmic filaments.

## Discussion

"Mesothelial/monocytic incidental cardiac excrescence" was firstly named by Veinot et al in 1994[1]. In the present case, the vegetation consisted of two populations of cells: histiocytoid cells and epithelial-like cells, with the former being predominant. The ''epithelial'' tissues arranged in glandular-like structure and were positive for cytokeratin AE1/AE3, cytokeratin 5/6, calretinin, and WT-1. Moreover they contained desmosomes, and numerous long slender microvilli in ultra- structure. Both immunohistochemistry and ultrastructure demonstrated that the ''epithelial'' tissues were indeed mesothelial cells. The major components were histiocytes which showed immnunoreactivity for CD68. The solid growth pattern of MICE may lead to consideration of various neoplasms such as malignant mesothelioma, metastatic carcinoma, ectopic meningioma, and paraganglioma. In this case, paraganglioma was initially considered before immunohistochemical studies were performed. However, the cells were negative for synaptophysin, chromogranin, and S100 proteins with the immunostain, denying its origins from neuroendocrine cells.

Until recently, 40 cases of MICE [2-5], including our case, have been reported. Of these, there were 21 males and 19 females, which indicates that MICE appears equally distributed between males and females. The age ranged from 5 to 80 years and most of the cases involved individuals above 60 years of age. The lesions ranged in size from microscopic to 3.5 cm. Nineteen of 40 cases (47.5%) underwent surgery for mitral valve repair, followed by atrial valve replacement (6/40, approximately 15%). The investigated specimens were mainly obtained from left atrium, mitral valve, pericardial sac and right ventricle. Other sites included right atrium, ascending aorta, right appendage, left atrioventricular groove and celiac artery.

To date, two theories, 'reactive'' theory and "artifact" theory, have been proposed to explain how the mesothelial cells ''gain'' access into the intravascular compartment. The so-called ''reactive'' theory stated that mesothelial cells migrate through cardiac wall at site of perforation during cardiac catheterization. This was supported by many reports which reported that the majority of patients had cardiac catheterization previously. However, the "artifact" hypothesis postulated that MICE are artifactual, formed by the manipulation of the cardiac surgeon, compacted by suction vacuum at the time of the procedure. Courtice et al demonstrated that materials from the surgical pump filters and drains were similar to MICE [6]. This mechanism theoretically could explain the reported case in which no prior catheterization was performed, but can not explain cases detected by endomyocardial biopsy without prior cardiac surgery. No prior catheterization was preformed in our case. So the MICE could not be formed by the perforation/ingrowth mechanism. In addition, once the pericardial sac was opened, the free floating vegetation was found by surgeon immediately, we can eliminate the possibility of iatrogenesis. Besides our case, the two hypotheses face many challenges, and they could not explain the reason that metastatic adenocarcinoma was accompanied with an obvious cardiac MICE in pericardial sac [7]. Although there are sound evidences for both theories of how MICE is formed, neither the 'reactive' nor 'artifactual' theory is sufficient to explain all the reported cases. We believe that MICE may be just a reactive lesion which not only results from cardiac catheterization, but also from inflammation, mechanic or tumor stimulation. Our case may be attributed to the inflammatory irritation of rheumatic pericarditis. Nodular histiocytic/mesothelial hyperplasia (NHMH), a similar entity to MICE, is also predominantly composed of histiocytes with scattered mesothelial cells [8,9]. Such processes can occur in the pericardium, pleura, peritoneum, and pelvis [10]. NHMH may be also a reactive lesion which could result from imflammation, mechanic irritation, or tumor.

Based on their similar morphologic features and the fact that the term MICE does not exactly reflect the essence of this entity, we propose to unify MICE and NHMH and NHMH may be a better choice.

In addition, awareness of this lesion by pathologist will avoid misdiagnosis and will obviate unnecessary invasive procedures or potentially dangerous therapies.

## Conclusion

MICE is a rare entity which is an amalgam of mesothelial cells, histiocytes, and fibrin. NHMH is a similar lesion to MICE. MICE and NHMH could be unified, and NHMH may be a better choice.

## Consent

Written informed consent was obtained from the patient for publication of this case report and any accompanying images. A copy of the written consent is available for review by the Editor-in-Chief of this journal.

## Competing interests

The authors declare that they have no competing interests.

## Authors' contributions

ZLH and OJ participated in the pathological examination of the case,the design of the study and drafting the manuscript. HL participated in the ultrastructure analysis. HLY participated in the pathological examination of the case. JFW participated in its design and coordination and helped to draft the manuscript.

All authors have read and approved the final manuscript.
